# Identification of Crucial Hub Genes and Differential T Cell Infiltration in Idiopathic Pulmonary Arterial Hypertension Using Bioinformatics Strategies

**DOI:** 10.3389/fmolb.2022.800888

**Published:** 2022-01-20

**Authors:** Xiaomei Yang, Cheng Wang, Yicheng Lin, Peng Zhang

**Affiliations:** ^1^ Department of Anesthesiology, Qilu Hospital, Cheeloo College of Medicine, Shandong University, Ji’nan, China; ^2^ School of Medicine, Cheeloo College of Medicine, Shandong University, Ji’nan, China; ^3^ Department of Neurology, Shandong Qianfoshan Hospital, Cheeloo College of Medicine, Shandong University, Ji’nan, China

**Keywords:** idiopathic pulmonary arterial hypertension, bioinformatics methods, differentially expressed genes, hub genes, immune infiltration

## Abstract

**Background:** Idiopathic pulmonary arterial hypertension (IPAH) is a life-threatening disease. Growing evidence indicated that IPAH is a chronic immune disease. This study explored the molecular mechanisms and T cell infiltration of IPAH using integrated bioinformatics methods.

**Methods:** Gene expression profiles of dataset GSE113439 were downloaded from the Gene Expression Omnibus and analyzed using R. Protein-protein interaction (PPI) network and gene set enrichment analysis (GSEA) were established by NetworkAnalyst. Gene Ontology enrichment analysis was performed using ClueGO. Transcription factors of differentially expressed genes (DEGs) were estimated using iRegulon. Transcription factors and selected hub genes were verified by real-time polymerase chain reaction (qPCR) in the lung tissues of rats with pulmonary artery hypertension. The least absolute shrinkage and selection operator regression model and the area under the receiver operating characteristic curve (AUC) were applied jointly to identify the crucial hub genes. Moreover, immune infiltration in IPAH was calculated using ImmuCellAI, and the correlation between key hub genes and immune cells was analyzed using R.

**Results:** A total of 512 DEGs were screened, and ten hub genes and three transcription factors were filtered by the DEG PPI network. The DEGs were mainly enriched in mitotic nuclear division, chromosome organization, and nucleocytoplasmic transport. The ten hub genes and three transcription factors were confirmed by qPCR. Moreover, *MAPK6* was identified as the most potent biomarker with an AUC of 100%, and ImmuCellAI immune infiltration analysis showed that a higher proportion of CD4-naive T cells and central memory T cells (Tcm) was apparent in the IPAH group, whereas the proportions of cytotoxic T cells (Tc), exhausted T cells (Tex), type 17 T helper cells, effector memory T cells, natural killer T cells (NKT), natural killer cells, gamma-delta T cells, and CD8 T cells were lower. Finally, MAPK6 was positively correlated with Tex and Tcm, and negatively correlated with Tc and NKT.

**Conclusion:** *MAPK6* was identified as a crucial hub gene to discriminate IPAH from the normal group. Dysregulated immune reactions were identified in the lung tissue of patients with IPAH.

## Introduction

Idiopathic pulmonary arterial hypertension (IPAH), also termed primary pulmonary hypertension, is a rare disease characterized by narrowing, and obliterating pulmonary vessels (Matsubara and Ogawa, 2014). Despite improvements in multimodal therapies, including supportive therapy, drug treatment, surgery, and transplantation, IPAH has a poor prognosis, and its overall 5-years survival rate remains low (Galie et al., 2016; Ogawa et al., 2017). The mean period from the onset of dyspnea symptoms to diagnosis among patients with PAH in the National Institutes of Health registry is approximately 2 years (Taichman and Mandel, 2013). The median survival time of patients is 2.8 years without PAH drug treatment (Rich et al., 1987). Therefore, there is an urgent need to discover reliable biomarkers for early diagnosis, prognosis evaluation, and targeted treatment for the disease.

Multiple mechanisms are involved in the remodeling of pulmonary arteries (Thompson and Lawrie, 2017). However, the findings related to PAH are inconsistent, and specific targets for the diagnosis and treatment of PAH require confirmation (Maron and Galie, 2016). Chronic inflammation and immune responses are important driving forces in IPAH (Soon et al., 2010). However, few studies have explored the infiltration of different immune cell subsets in IPAH. Bioinformatics data mining of gene expression microarray data provided by the Gene Expression Omnibus (GEO) database is convenient and helpful in identifying potential genes involved in the pathogenesis of diseases and reveals valuable insights for future research (Kulasingam and Diamandis, 2008). The ImmuCellAI online software provides the opportunity to explore the differential expression patterns of immune cell infiltration in diverse samples using microarray data (Miao et al., 2020). Comprehensive analyses of immune infiltration and biomarkers using bioinformatics methods are helpful to explore the molecular mechanisms of IPAH and to diagnose it accurately. In this study, we used the ImmuCellAI algorithm to calculate the relative compositions of different T cells in IPAH samples and normal samples.

In the present study, the expression profile GSE113439, deposited by M. Mura, was re-analyzed to identify critical genes using extensive bioinformatics methods, including differential analysis, protein-protein interaction (PPI) network construction, transcription factor (TF)-differentially expressed genes (DEGs) network construction, and crucial hub gene identification. Based on the GEO microarray data, we compared the differential infiltration of T cell subtypes in IPAH lung tissue and normal controls, and explored the relationship between key hub genes and different immune cell subsets. The hub genes and TFs were confirmed in a PAH rat model induced by monocrotaline.

## Methods

### Data Collection and DEG Identification

The GSE113439 dataset of PAH with clinical manifestation data was downloaded from the GEO database (https://www.ncbi.nlm.nih.gov/geo/). The platform was GPL6244 (Affymetrix Human Gene 1.0 ST Array), submitted by M. Mura on April 20, 2018 (Mura et al., 2019). Six patients had IPAH, and 11 were normal controls. The raw data files were read using the “affy” package in R (version 3.5.1; R Project of Statistical Computing, Vienna, Austria) and then normalized using the robust multi-array average algorithm. The probes were annotated with annotation files, and probes without corresponding gene symbols were removed. The “limma” package in R was used to identify DEGs between patients with IPAH and normal controls. The fold change (FC) in gene expression was calculated, and |log2FC| ≥ 1 and *p*-values < 0.05 were set as the cut-off criteria.

### Gene Set Enrichment Analysis

Gene set enrichment analysis (GSEA) was performed using NetworkAnalyst, a visual analysis platform for comprehensive gene expression profiling and meta-analysis (https://www.networkanalyst.ca/) (Zhou et al., 2019). The expression matrix of all genes and phenotypes of the samples were uploaded to the “gene expression table” input area and analyzed in a streamlined manner. Pathway enrichment analysis was performed using the normalized enrichment scale (NES), and pathways that were upregulated or downregulated in the IPAH samples, compared with normal samples, and were determined.

### Functional Annotation and Gene Ontology Analysis

To evaluate the biological functions of the proteins in the PPI networks of the total DEGs, we used the ClueGO app in Cytoscape (version 3.7.1, http://www.cytoscape.org/) to interpret and visualize the over-represented Gene Ontology (GO) terms for protein members in the network. The ClueGO app in Cytoscape integrates GO terms into a PPI network and creates a functional annotation map that represents the associations and interactions in the network. A κ score of 0.4 and a *p*-value < 0.05 were set as the cutoff criteria (Mlecnik et al., 2019).

### PPI Network Construction

The STRING app in NetworkAnalyst was used to construct the PPI network. According to the interaction pair information, the PPI network was created, and visualized using the Cytoscape software. Hub genes with a high degree of connectivity were selected from the PPI network, with a connectivity degree >80 and lung-specificity as the cutoff criteria. Using the search tool for the retrieval of interacting genes (STRING) (www.string-db.org) online tool (Szklarczyk et al., 2021), we predicted the associations between proteins coded by the hub genes.

### Construction of TF-DEG Regulation Networks

The iRegulon app in Cytoscape was used to identify the TFs that target the DEGs. The enrichment of TF motifs in iRegulon was based on direct targets using the position weight matrix method (Janky et al., 2014). In this study, the prediction of TFs was estimated according to the following parameters: the minimum identity between orthologous genes was 0.05, the maximum false discovery rate on motif similarity was equal to 0.001, and NES >4 was set as the threshold. The TF-DEGs network was constructed and visualized using Cytoscape.

### Regression Analyses

The least absolute shrinkage and selection operator (LASSO) regression was used to select the most significant variables. The expression of ten hub genes was analyzed using LASSO regression with a binomial model and lambda value equal to the minimum mean cross-validated error. The “glmnet” package in R was used to predict the most likely hub genes. Then, the area under the receiver operating characteristic (ROC) curve (AUC) was constructed using the “pROC” package in R and used to confirm the LASSO regression analysis. The correlation between the candidate hub genes and IPAH was analyzed using Pearson correlation analysis with the “corrplot” package in R.

### Immune Cell Infiltration Analysis

Normalized gene expression data were uploaded to the ImmuCellAI online website, and 24 types of immune cell infiltration were estimated in the two groups using online software. The proportion of the 24 types of immune cells in every sample was verified using the “tidyr” package in R and visualized using the “ggplot2” package in R. Immune cells in the ImmuCellAI analysis (*p* < 0.05) were selected for the correlation analysis. Pearson correlation analysis was used to construct the correlation of significant immune cells and crucial hub genes using the “corrplot” R package.

### Pulmonary Arterial Hypertension Animal Model

Twelve male Sprague-Dawley rats (220–320 g) were used in this study (*n* = 6 per group). Monocrotaline was injected intraperitoneally at a dose of 60 mg/kg on day 1 of the experiment. Six control animals were injected intraperitoneally with normal saline. The animals were housed at the Animal Care Facility of Shandong University until day 14 of the experiment at an ambient temperature of 22°C with a 12-h light/dark cycle. All animals received standard rodent food and water ad libitum. The experimental protocol was approved by the Medical Ethics Committee for Experimental Animals of Shandong University (number ECAESDUSM 2012029).

### Hematoxylin-Eosin Staining and Immunohistochemistry

Rat lung tissues were fixed in 4% paraformaldehyde for 24 h, dehydrated, cleared, embedded in paraffin wax, sliced into 5-μm-thick sections, and stained with hematoxylin and eosin. For immunohistochemistry, the lung tissues were dewaxed and incubated overnight with primary antibodies at a dilution of 1:1,000, followed by three washes with phosphate-buffered saline, and incubation using the SPlink Detection Kit (Biotin-Streptavidin HRP Detection System; ZSGB-BIO, Beijing, China). Following incubation, the tissue sections were stained with 3,3-diaminobenzidine and re-stained with hematoxylin. Images were captured using a fluorescence microscope (Nikon-C-SHG1, Tokyo, Japan) equipped with a digital camera. Muscular arteries with diameters <100 μm per lung section were outlined and measured.

### Real-Time Polymerase Chain Reaction Analysis

The relative levels of target genes were measured using real-time polymerase chain reaction (qPCR). Total RNA from heart tissues was extracted using the RNeasy Fibrous Tissue Mini Kit (QIAGEN, Hilden, Germany) according to the manufacturer’s instructions. cDNA was synthesized using the High Capacity cDNA Reverse Transcription Kit (Thermo Fisher Scientific, Waltham, MA). Fluorescent quantification of target genes was performed using the ABI 7500 fast real-time PCR system (Applied Biosystems, Foster City, CA) with a 20-μl reaction mixture volume that included the following: cDNA 1 μL, forward and reverse primers 2 μl, Fast SYBRGreen Master Mix 10 μl (Thermo Fisher Scientific), and nuclease-free water 7 μl. The primers used for this process are listed in Table 1. Primers were obtained from Integrated DNA Technologies (Coralville, IA). Relative mRNA levels were normalized to those of glyceraldehyde 3-phosphate dehydrogenase.

**TABLE 1:** Sequences of RT-PCR primers.

Gene	Primer F (5′-3′)
HSP90AA1	GTT​TCG​TGC​GTG​CTC​ATT​CT
CDC5L	CGT​GTG​GAG​GAA​TAC​GGA​GG
LRRK2	TCA​ATA​GCA​AGC​GAG​CGA​CT
APC	TAT​GCG​CCC​AAG​TCC​TTT​CA
HNRNPA1	GGG​GAT​TTG​CGT​TTG​TCA​CC
IQGAP1	CTC​ACC​ACA​GAC​CAG​CGT​AG
MET	AGT​CCT​ACA​TTG​ATG​TCC​TGG​GAG
PIK3R1	CAT​CGA​CCT​ACA​CTT​GGG​GG
MAPK6	GAG​TCG​GAG​AAG​TCC​CGT​TG
HIF1A	GCA​ACT​GCC​ACC​ACT​GAT​GA
GATA3	ATG​GAG​GTG​ACT​ACG​GAC​CA
TAF1	TCA​GAA​TGT​TGA​ATG​AAG​ACA​GAA​A
RBBP9	ACC​AAG​GCA​GTG​ATT​GTT​CC

RT-PCR, Real-time polymerase chain reaction.

### Statistical Analyses

All statistical analyses were performed using online software and the corresponding packages in R. One-way analysis of variance was used for the statistical analysis, and *p <* 0.05 indicated a significant difference.

## Results

### Identification of DEGs

**FIGURE 1:**
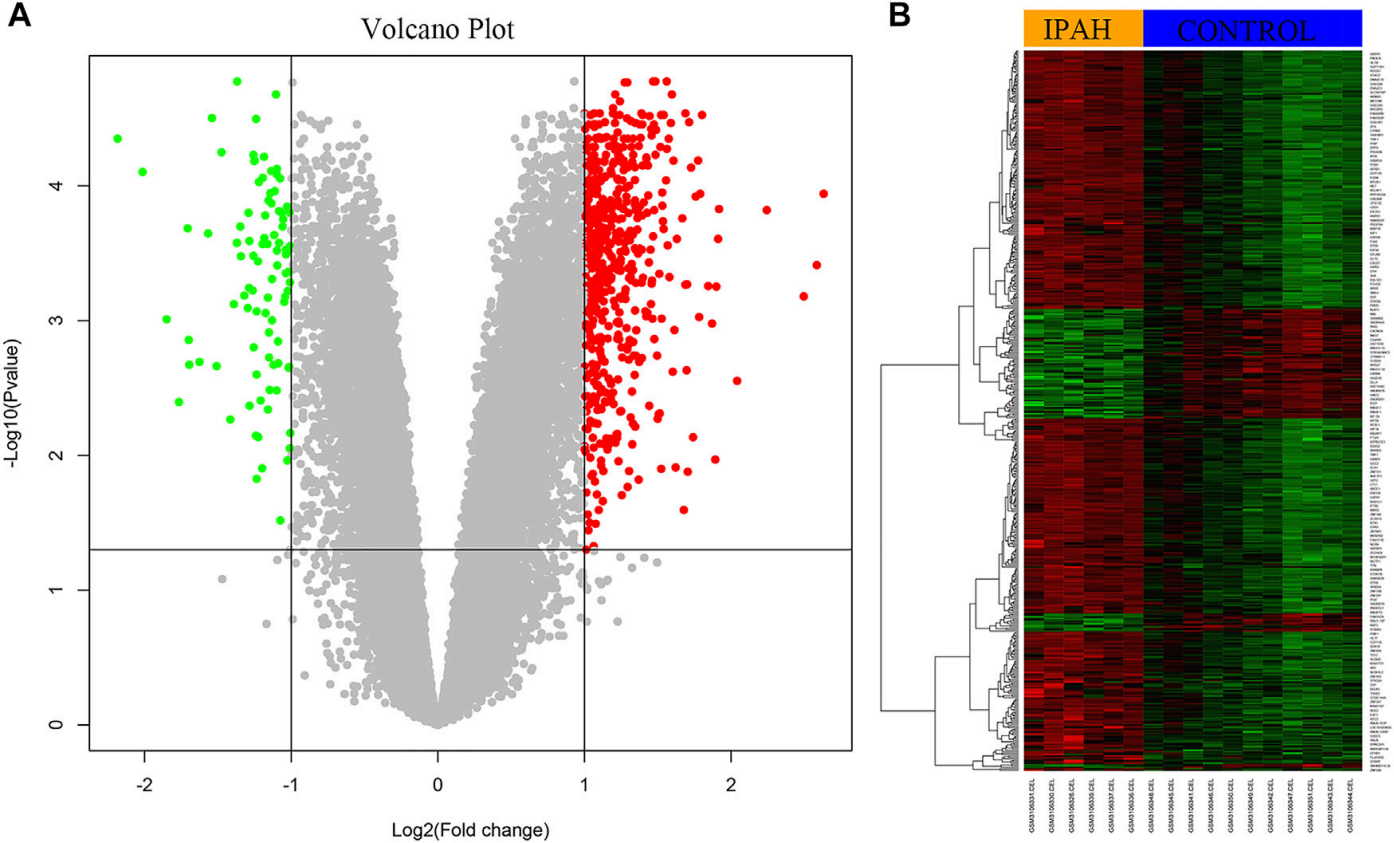
Identification of DEGs from dataset GSE113439. **(A)** The volcano plot of DEGs from six IPAH samples and 11 control samples. The red color represents upregulation and the green color represents down-regulation of genes. **(B)** The heat map of upregulated and downregulated DEGs. Red color represents upregulation and green color represents downregulation of genes. DEGs, differentially expressed genes; IPAH, idiopathic pulmonary artery hypertension.

Using |log2FC| ≥ 1 and *p* < 0.05, the GSE113439 dataset, including six IPAH samples and eleven control samples, and was analyzed using R. In total, 512 DEGs were identified, of which 419 were upregulated genes, and 93 were downregulated genes. The volcano plots of the DEGs are shown in Figure 1A. Each colored dot represents an up- or down-regulated gene; green dots indicate genes with a low level of expression, and red dots indicate genes with a high level of expression. Moreover, gray dots indicate genes with no change or differential expression based on the criteria of |log2FC| ≥ 1 and *p* < 0.05. Heatmap analysis showed that DEGs were correctly distinguished between the IPAH and control samples (Figure 1B).

### Gene Set Enrichment Analysis

**FIGURE 2:**
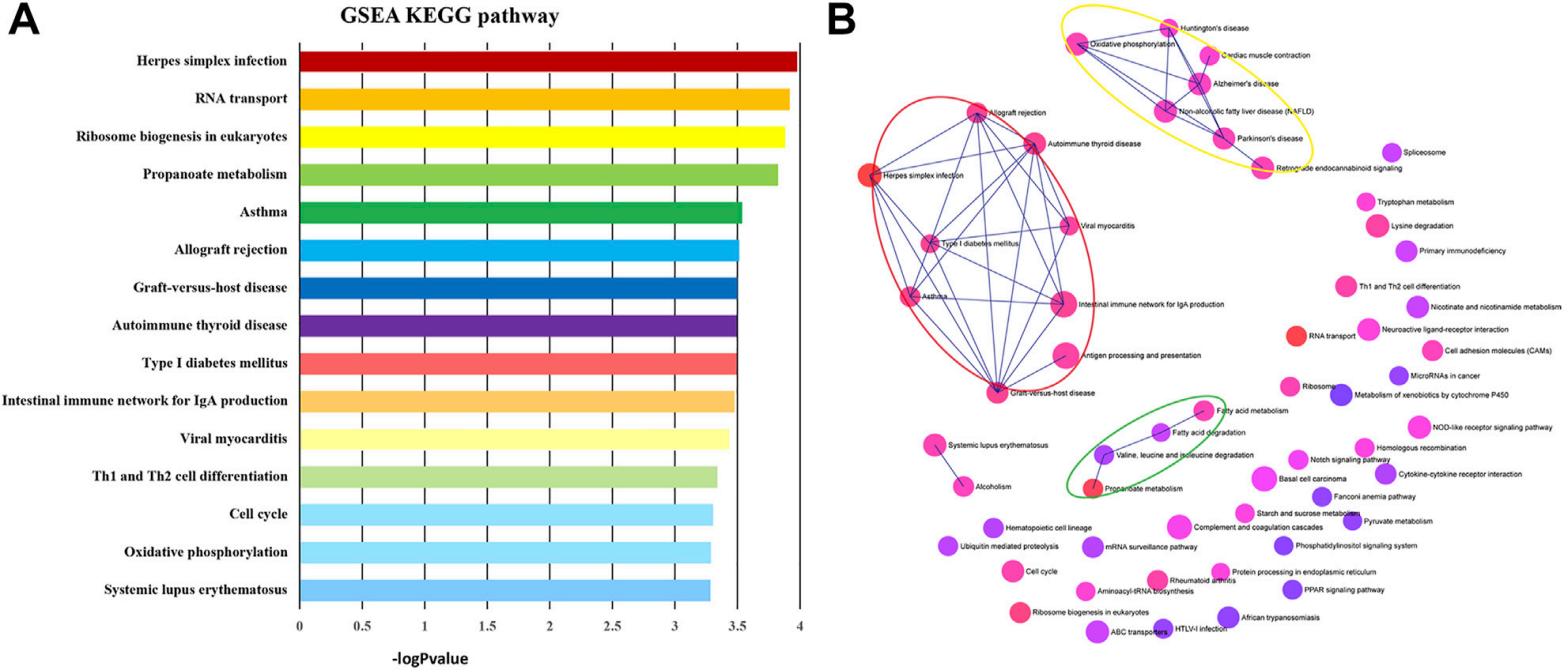
KEGG pathways of GSEA. **(A)** The most significantly enriched pathway terms of GSEA KEGG pathways. **(B)** The network of GSEA KEGG pathways. KEGG, Kyoto Encyclopedia of Genes and Genomes; GSEA, gene set enrichment analysis.

GSEA Kyoto Encyclopedia of Genes and Genome (KEGG) pathway analysis was performed to discover crucial biological pathways and potential molecular mechanisms using the genome-wide expression profiles of IPAH. According to the ranking and running enrichment scores, the top 15 results for the most significant pathways are shown in Figure 2A, and the relationships of the different pathways are shown in Figure 2B.

### GO Function Analysis

**FIGURE 3:**
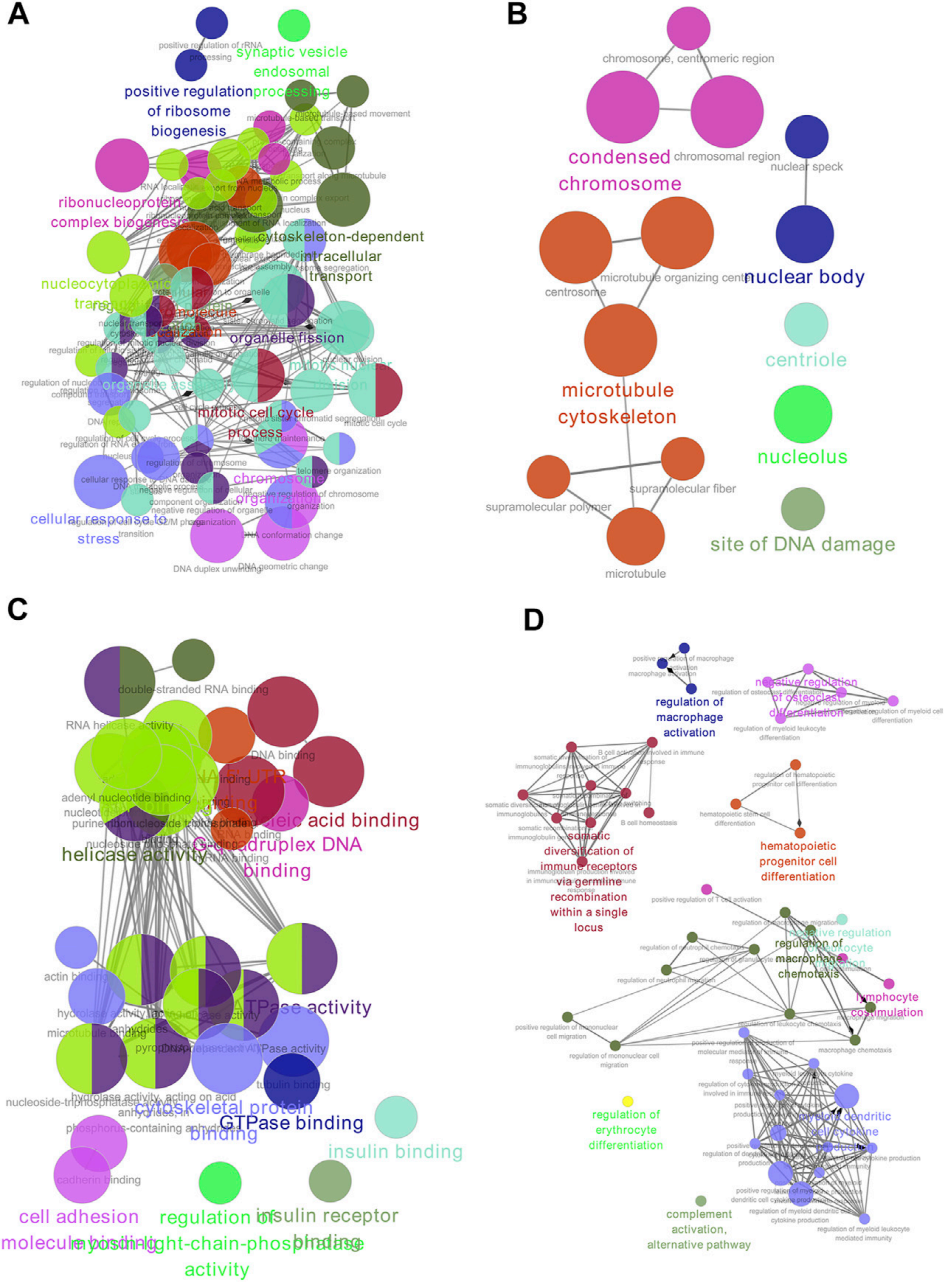
GO enrichment of DEGs. **(A)** Biological process. **(B)** Cell component. **(C)** Molecular function. **(D)** Immune system analysis. GO, gene ontology; DEGs, differentially expressed genes.

GO functional enrichment analysis and functional annotation of candidate DEGs were performed using the ClueGO app (Figures 3A–D). For biological processes, the DEGs were mainly enriched in mitotic nuclear division, chromosome organization, nucleocytoplasmic transport, and cellular macromolecule localization (Figure 3A). For the cellular component, the DEGs were primarily enriched in the microtubule cytoskeleton, condensed chromosome, and nuclear body (Figure 3B). For molecular function, the DEGs were mainly enriched in ATP binding, ATPase activity, cytoskeletal protein binding, and helicase activity (Figure 3C). Finally, for immune system processes, the DEGs were enriched in myeloid dendritic cell cytokine production, regulation of macrophage chemotaxis, and somatic diversification of immune receptors via germline recombination within a single locus (Figure 3D).

### PPI Network Construction

**FIGURE 4:**
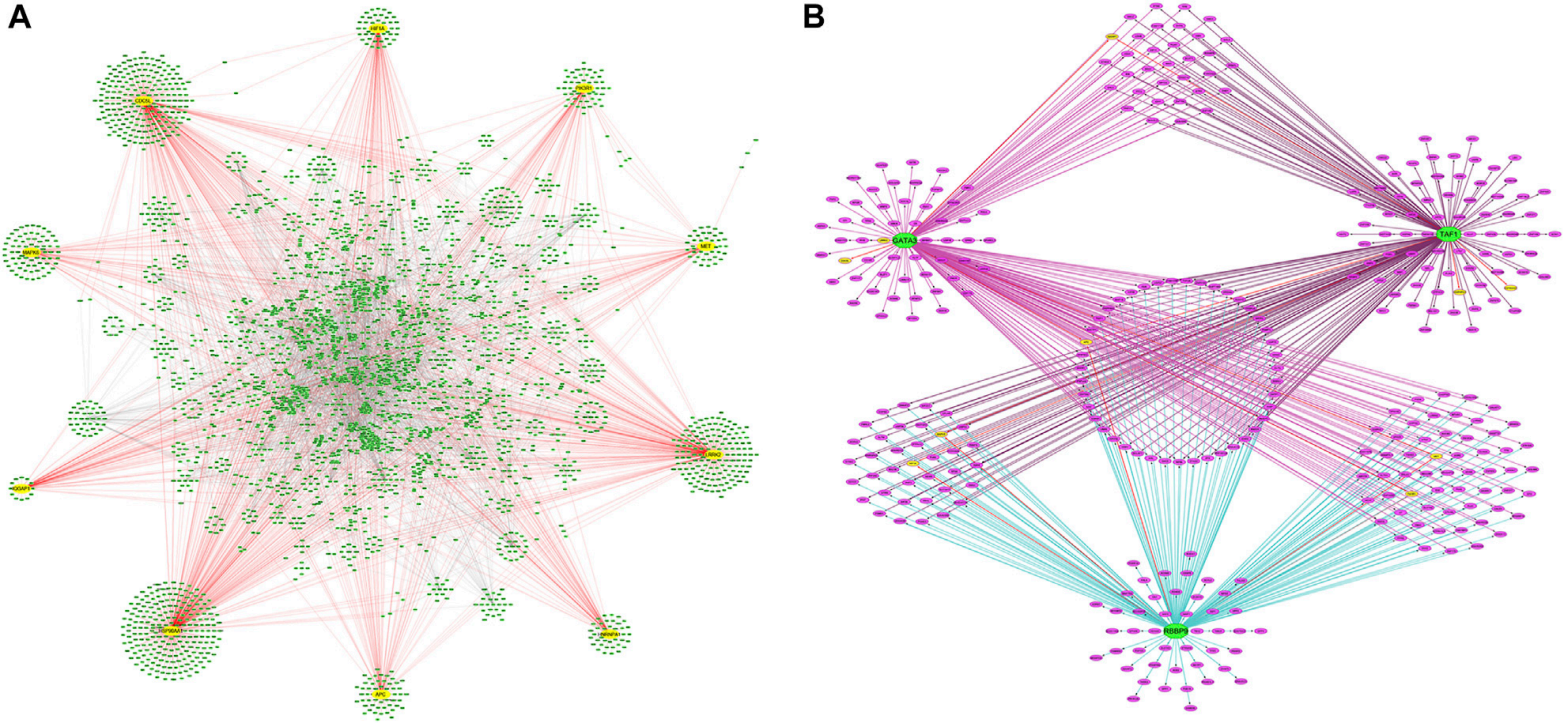
PPI networks of DEG and TFs-DEGs networks. **(A)** The PPI network of DEGs. Yellow circles are hub genes. **(B)** Network of predicted TFs and DEGs. Green circles are TFs, yellow circles are hub genes, and purple circles are DEGs. PPI, protein-protein interaction; TFs, transcriptional factors; DEGs, differentially expressed genes.

The candidate DEGs were uploaded into the STRING app in NetworkAnalyst, and the results are presented as a PPI network visualized by Cytoscape (Figure 4A). A total of 341 seeds from 512 candidate DEGs were filtered into a network consisting of 5,950 interaction pairs among 3,263 nodes. According to the network topology parameters, ten genes with higher degrees of connectivity were identified as hub genes: heat shock protein 90 alpha family class A member 1 (*HSP90AA1*, degree = 439), cell division cycle 5-like protein (*CDC5L*, degree = 396), leucine-rich repeat serine/threonine-protein kinase 2 (*LRRK2*, degree = 331), phosphatidylinositol-3-kinase regulatory subunit alpha (*PIK3R1*, degree = 120), mitogen-activated protein kinase 6 (*MAPK6*, degree = 116), hypoxia-inducible factor 1 alpha (*HIF1A*, degree = 116), adenomatous polyposis coli (*APC*, degree = 114), heterogeneous nuclear ribonucleoprotein A1 (*HNRNPA1*, degree = 110), receptor tyrosine kinase (*MET*, degree = 94), and IQ motif-containing GTPase-activating protein 1 (*IQGAP1*, degree = 93) (Figure 4A).

### TF Regulatory Network Analysis of DEGs

To further understand the regulatory network between TFs and DEGs, the iRegulon plugin in Cytoscape was used to explore the TFs of the DEGs of interest. Three TFs were identified with an NES >4 in the gene-TF regulatory network: retinoblastoma-binding protein 9 (RBBP9), GATA binding protein 3 (GATA3), and TATA-box-binding protein-associated factor 1 (TAF1). One-hundred-and-eighty-two DEGs were predicted as targets of RBBP9, 186 DEGs as targets of GATA3, and 188 DEGs as potential targets of TAF1. Forty-three DEGs, including the hub gene *APC*, were co-regulated by GATA, TAF1, and RBBP9. The Hub gene *IQGAP1* was regulated by GATA3 and TAF1. Hub genes *MAPK6* and *HIF1A* were regulated by TAF1 and RBBP9, respectively, whereas *MET* and *PIK3R1* were individually regulated by GATA3 and RBBP9 (Figure 4B).

### Regression Analyses

**FIGURE 5:**
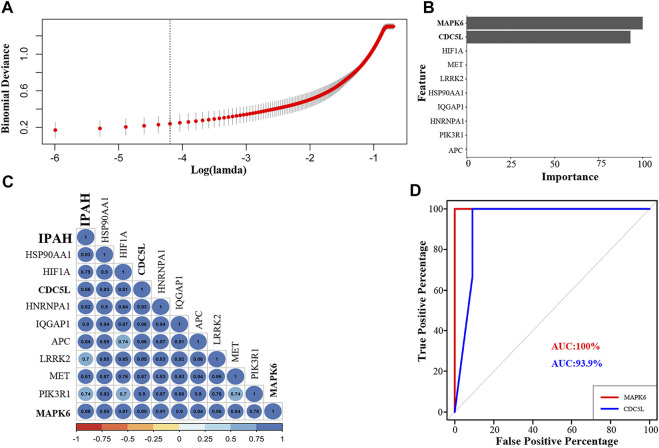
Regression analysis, correlation analysis, and AUC analysis for the prediction of crucial hub genes in IPAH patients. **(A)** Log(lambda) of LASSO. **(B)** LASSO regression results of ten hub genes. **(C)** Correlation of hub genes with IPAH **(D)**. The AUC of the multiple receiver operating characteristic curves for MAPK6 and CDC5L. LASSO, least absolute shrinkage and selection operator; AUC, area under the receiver operating characteristic curve; IPAH, idiopathic pulmonary artery hypertension.

To identify the best hub genes for IPAH, we used LASSO regression to analyze the ten hub genes. Two genes, *MAPK6* and *CDC5L*, were filtered using binomial regression (Figures 5A,B). The Pearson correlation coefficients of *MAPK6* and *CDC5L* with IPAH were 0.88 (Figure 5C). The predictive value of *MAPK6* was evaluated by ROC curve analysis with an AUC of 100% (Figure 5D). *MAPK6* was regarded as the most likely hub gene overlapped by the three algorithms.

### Immune Cell Infiltration Analysis

**FIGURE 6:**
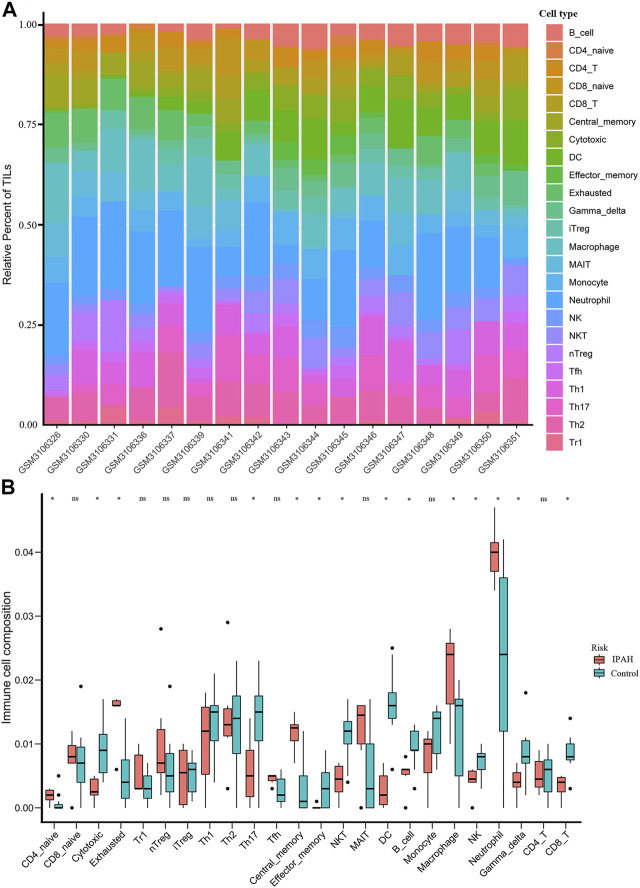
Immune cells infiltration predicted via ImmuCellAI. **(A)** Composition of 24 Immune cell subsets generated by ImmuCellAI in 17 samples from the GSE113439 dataset. **(B)** Infiltration of different immune cells between IPAH and normal controls. Red box represents the IPAH group; blue box represents the normal group. (*p < 0.05). IPAH, idiopathic pulmonary artery hypertension.

The relative proportions of the 18 subtypes of T cells and six other immune cells among the IPAH and control samples were estimated using the ImmuCellAI algorithm (Figure 6A). Fourteen types of immune cells were significantly different between the IPAH and control groups (Figure 6B). Among the T cell subtypes, CD4 naive T cells (*p* = 0.049), exhausted T cells (*p* = 0.0046) and central memory T cells (*p* = 0.0031) occurred at higher levels in the IPAH group than in the control group, whereas the levels of cytotoxic T cells (*p* = 0.01), type 17 T helper cells (*p* = 0.03), effector memory T cells (*p* = 0.049), natural killer T (NKT) cells (*p* = 0.01), gamma-delta T cells (*p* = 0.03), and CD8 T cells (*p* = 0.01) were higher in the control group. Among the distinct IPAH-infiltrating non-T cells, the fractions of dendritic cells (*p* = 0.0018), B cells (*p* = 0.01), and natural killer cells (*p* = 0.02) were negatively associated with the IPAH group and positively associated with the normal group. Additionally, the fractions of macrophages (*p* = 0.049) and neutrophils (*p* = 0.02) were significantly higher in the IPAH group.

### Correlation Analysis

**FIGURE 7:**
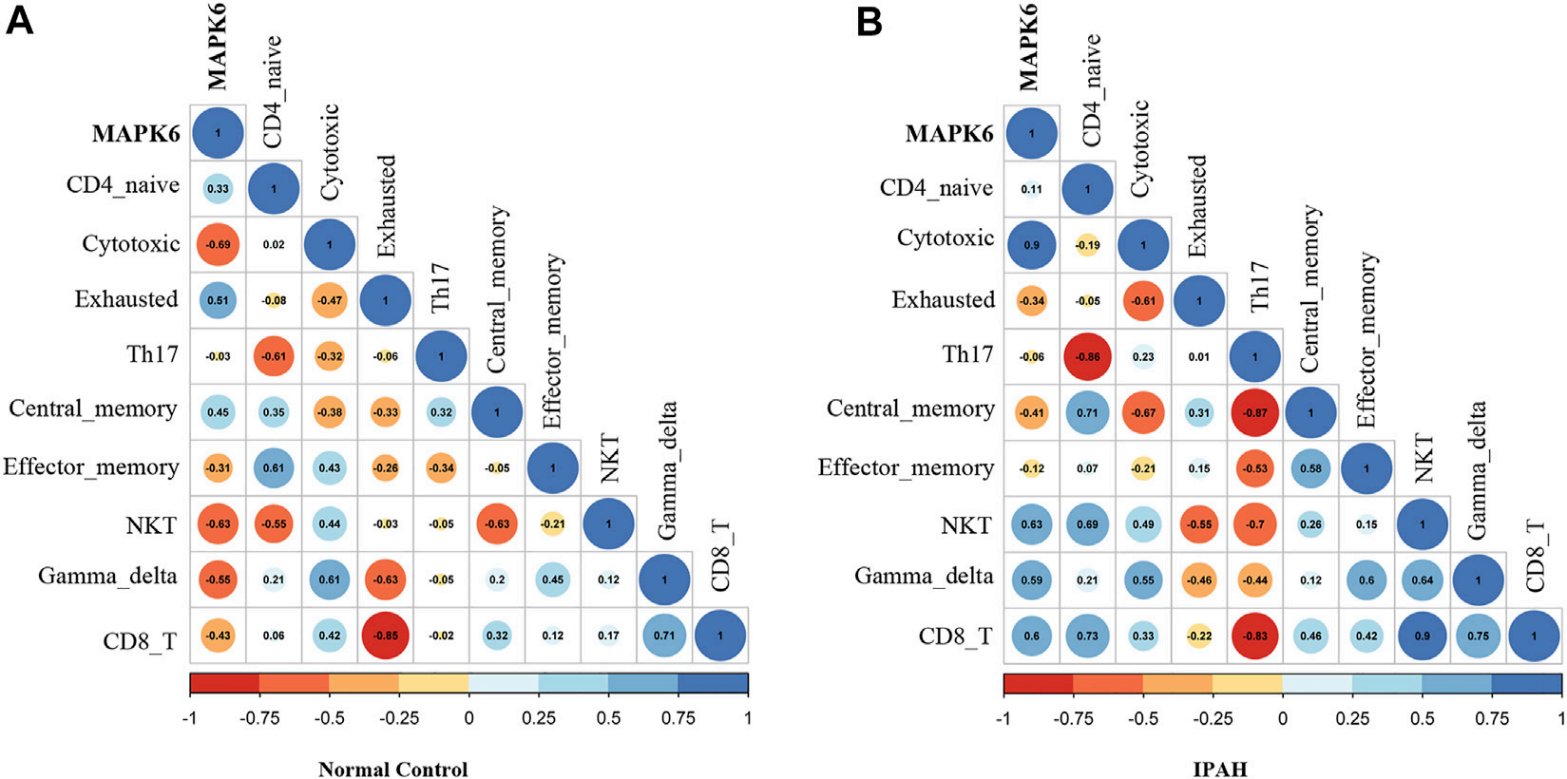
Correlation analysis. **(A)** Correlation of MAPK6 with significant T cell subtypes in the normal group. **(B)** Correlation of MAPK6 with significant T cell subtypes in the IPAH group. IPAH, idiopathic pulmonary artery hypertension.

The correlation between *MAPK6* and significant T cells in all samples is shown in Figure 7. It was demonstrated that *MAPK6* is positively correlated with exhausted T cells (R = 0.51) and central memory T cells (R = 0.45), and negatively correlated with cytotoxic T cells (R = −0.69) and NKT cells (R = −0.63) in the control group (Figure 7A). Whereas MAPK6 is positively correlated with cytotoxic T cells (R = 0.9) and NKT cells (R = 0.63), and negatively correlated with exhausted T cells (R = −0.34) and central memory T cells (R = −0.41) in the IPAH group (Figure 7B).

### qPCR Validation of the Hub Genes and TFs

**FIGURE 8:**
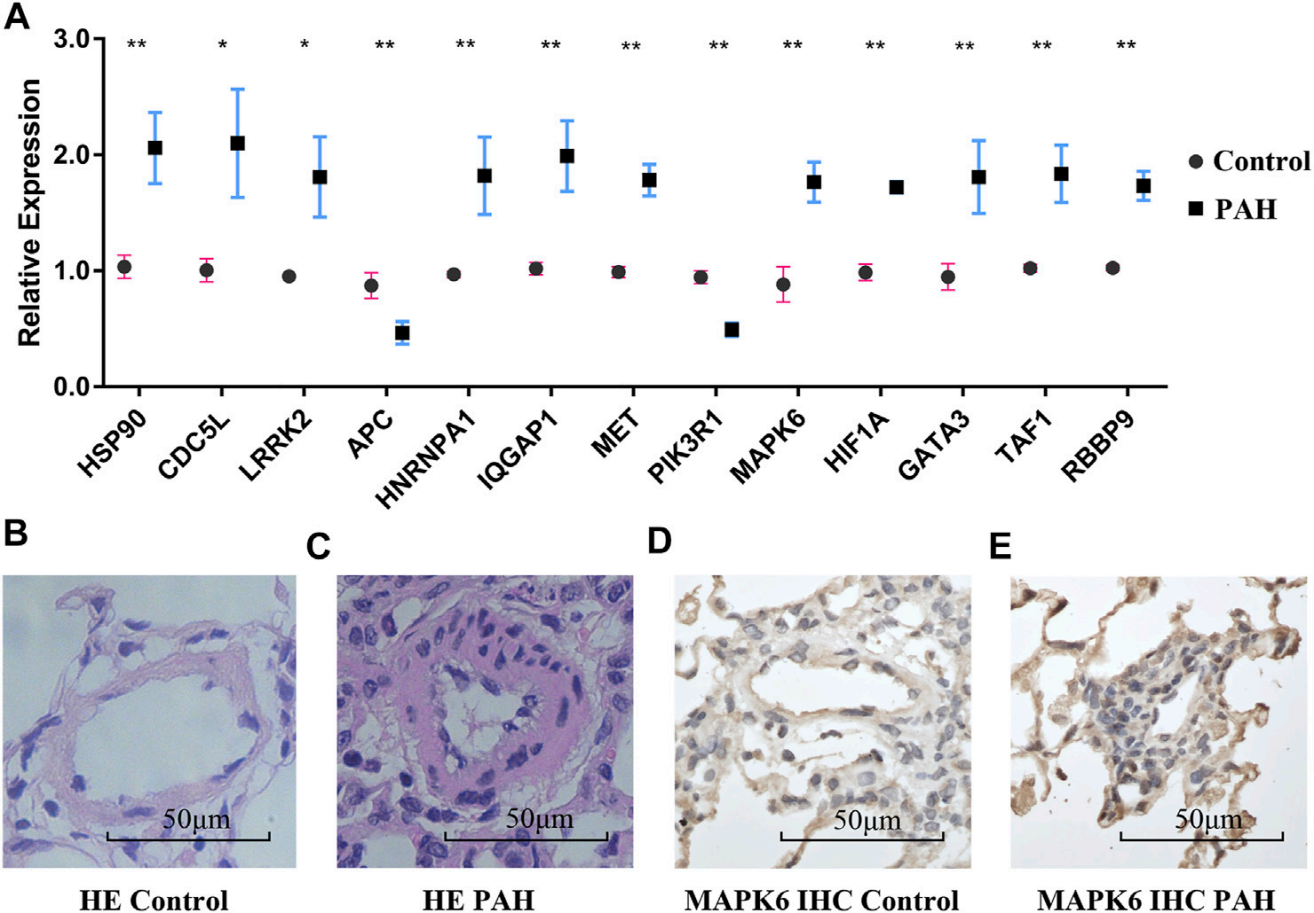
RT-PCR validation and pulmonary microarteries HE and IHC staining. **(A)** The mRNA expression of hub genes and TFs in the normal group and the IPAH group. **(B,C)** Pulmonary microarteries HE staining in the normal group and the IPAH group. **(D,E)** MAPK6 IHC staining of pulmonary microarteries in the normal group and the IPAH group. RT-PCR, Real-time polymerase chain reaction; TFs, transcriptional factors; HE, hematoxylin and eosin; IHC, immunohistochemistry; IPAH, IPAH, idiopathic pulmonary artery hypertension. (*p < 0.05).

The relative expressions of ten hub genes and three TFs are shown in Figure 8A. The hub genes *HSP90AA1*, *CDC5L*, *LRRK2*, *PIK3R1*, *MAPK6*, *HIF1A*, *HNRNPA1*, *MET*, and *IQGAP1,* and TFs RBBP9, GATA3, and TAF1 were significantly higher in PAH rat lung tissue after 2 weeks of monocrotaline injection.

### Hematoxylin-Eosin Staining and Immunohistochemistry

Hematoxylin-eosin staining of the pulmonary vessels is shown in Figures 8B,C. More immune cell infiltration was visible around the remodeled pulmonary vessels in the IPAH group than around the vessels of the normal controls. The expression of the hub gene *MAPK6* in the remodeled pulmonary vessels was higher than that in the normal pulmonary vessels (Figures 8D,E).

## Discussion

IPAH is characterized by occlusive vasculopathy of the pulmonary arteries (Firth et al., 2010), the underlying mechanism and immune infiltration involved in the remodeling of pulmonary arteries are not fully elucidated. Our study is the first to identify crucial hub genes and T cell subtype infiltration in IPAH using bioinformatics strategies. Of the 512 DEGs identified, 419 genes were upregulated, and 93 were downregulated. Ten hub genes were filtered from the PPI network according to the degree of connectivity and tissue specificity. Furthermore, ten hub genes and three TFs were verified in a PAH rat model. The relationship between the crucial hub genes and immune cells was revealed.

Our rat PAH model revealed obviously increased inflammatory cells around the remodeled pulmonary arterial vessels, similar to previous studies (Rabinovitch et al., 2014; Goldenberg et al., 2019; Tang et al., 2021). To further explore the comprehensive infiltration characteristics of immune cells in IPAH lungs, we calculated the immune cell subtypes and explored the differences in T cell subsets between IPAH and normal samples. Our results revealed that the two groups expressed different subtypes of immune cells. Among the T cell subtypes, increased expression of CD4-naive T cells, exhausted T cells, and central memory T cells was observed in the IPAH group compared with the normal controls, whereas the expression of other T cells, such as cytotoxic T cells, type 17 T helper cells, effector memory T cells, NKT, gamma-delta T cells, and CD8 T cells was lower in the lungs of IPAH patients.

The relationship between the immune system and the development of pulmonary hypertension has been explored for decades (Voelkel et al., 2016). It has been proven that T cell-deficient rats, not immune-reconstituted rats, develop more severe pulmonary hypertension after SU5416 injection (Tamosiuniene et al., 2011). However, no studies have clearly elucidated the roles of different T cell subtypes in pulmonary vascular remodeling in patients with IPAH. The increased expression and activation of CD4-naive T cells and central memory T cells have been associated with immune and inflammatory responses. During chronic viral infection, T cell exhaustion facilitates viral persistence, and exhausted T cells function restored by immune modulation promotes viral clearance (McKinney and Smith, 2016). Type 17 T helper cells defend against extracellular bacteria and fungi, and have been identified as having pro-inflammatory bias and play a critical role in autoimmune disorders (Tabarkiewicz et al., 2015). It is believed that type 17 T helper cells contribute to the development of PAH induced by chronic hypoxia (Maston et al., 2017). NKT cells have a cytolytic ability and release cytokines and chemokines to influence innate and acquired immune responses (Paget and Trottein, 2013). Reduced levels of NKT cells are believed to contribute to the development of systemic sclerosis, an autoimmune disease (Riccieri et al., 2005), and which is similar to the findings of this study. Gamma-delta T cells exert protective functions in barrier surveillance and first-line defense in lungs after influenza infection (Wang et al., 2021). Gamma-delta T cells were observed to be decreased in the blood of patients with asthma or *Bordetella pertussis* infection (Bertotto et al., 1997; Krejsek et al., 1998). Austin et al. revealed that there is a significant increase in the number of effector memory T cells in the peripheral blood of patients with IPAH versus controls and a prominent increase in the number of CD8 T cells in the lungs of patients with IPAH (Austin et al., 2010). While our study demonstrated a decrease of effector memory T cells and CD8 T cells in the lung of IPAH patients, decreased numbers and dysfunction of effector memory T cells and CD8 T cells usually indicate chronic viral infection (Barber et al., 2006; Kalia et al., 2010). The high risk of herpes simplex infection in the GSEA KEGG pathway supports the hypothesis that viral infection may be involved in IPAH. We hypothesized that the abnormal expression of T cell subsets in the IPAH lung might indicate a state of T cell impaired tolerance or exhaustion. It is unclear whether dysregulated adaptive immune cells promote or suppress pulmonary vessel remodeling in patients with IPAH. The relationship between altered T cell subtypes and virus infection in patients with IPAH is a topic for future research.

We also observed an accumulated number of macrophages and neutrophils, and a decreased number of dendritic and B cells in IPAH lungs. The accumulation and activation of macrophages and neutrophils are presumably involved in the remodeling of the pulmonary vasculature through cytokine and chemokine release (Hall et al., 2009). Accumulated dendritic and B cells were also identified in the remodeled pulmonary vessels of patients with IPAHs (Perros et al., 2007), unlike our research results, which showed a reduction in dendritic and B cells in the lungs of patients with IPAH. The reasons behind this reduction warrant further investigation. Therefore, our study provides some controversial information for the analysis of IPAH.

Whole-gene GSEA KEGG pathways were mainly enriched in herpes simplex infection, RNA transport, and the eukaryotic ribosome biogenesis-mediated signaling pathways. In our study, the herpes simplex infection pathway was associated with asthma, allograft rejection, type I diabetes mellitus, autoimmune thyroid disease, and graft-versus-host disease, implying the involvement of inflammatory and immune responses in IPAH development. Moreover, GO function enrichment of the total DEGs was mainly within mitotic nuclear division, chromosome organization, and nucleocytoplasmic transport. These biological process-related findings corroborated those of previous studies, which showed that abnormal proliferation and the resistance to apoptosis in pulmonary vascular remodeling were key pathological features of PAH (Sakao et al., 2009; Thompson and Lawrie, 2017). Immune system process enrichment revealed that myeloid dendritic cell cytokine production and macrophage chemotaxis regulation were present in the IPAH lung, suggesting that activated immune cells and cytokine release are involved in pulmonary vascular remodeling.

Precise transcriptional regulation of protein-encoding genes is critical for cell proliferation, differentiation, and development (Thomas and Chiang, 2006). TFs are considered to play vital roles in the development of pulmonary hypertension through pro-inflammatory, immunological, and multiple other cellular responses (Di Mise et al., 2017). To our knowledge, this is the first study to show that TFs RBBP9, GATA3, and TAF1 may be involved in the development of IPAH. RBBP9 is a tumor-associated protein that overcomes transforming growth factor β-1-induced growth arrest by binding to the retinoblastoma protein (Vorobiev et al., 2012). GATA3 belongs to the TF GATA family and exhibits diverse functions in regulating the immune response (Wan, 2014). TAF1, the largest subunit of the TF IID, is associated with the transcriptional regulation of a subset of essential genes such as G1 cyclins and major histocompatibility class I gene (Weissman et al., 1998; Dunphy et al., 2000). Our research revealed that the three TFs formed a connected regulatory network with the DEGs, suggesting that the dynamic changes in the activity of these TFs might play important roles in regulating the expression and function of DEGs associated with the occurrence and progression of IPAH.

Ten genes were identified as hub genes for the PPI network, and their expressions were verified in a PAH rat model induced by monocrotaline. *MAPK6*, also known as *ERK3*, was identified as a crucial hub gene by three methods of analysis. MAPK6 is involved in cell proliferation, differentiation, invasion, and immune response (Long et al., 2012; Marquis et al., 2014; Sirois et al., 2015; Tan et al., 2017). The expression of MAPK6 is required for thymic positive selection (Sirois et al., 2015). MAPK6 deficient T cells decrease proliferative capacity and secrete less cytokine (Marquis et al., 2014). Our research revealed that the MAPK6 expression was positively correlated with exhausted T cells and central memory T cells, and negatively correlated with type 17 helper T and NKT cells. Further research is needed to explore the role of MAPK6 in T cell activation and differentiation. Currently, there are no studies to corroborate our findings that the above-mentioned vital hub genes are critical to the development of IPAH, and our research may reveal valuable insights into the molecular mechanisms and diagnosis of IPAH.

This study had some limitations. First, we explored the functions and potential roles of hub genes without analyzing other DEGs. Second, there were no follow-up experiments to validate the roles of the hub genes and TFs in IPAH remodeling. Third, the roles of different T cell subsets in pulmonary vascular remodeling need to be verified in future research. Despite these limitations, our research provided novel findings in the study of IPAH and laid the groundwork for further examination of this disease and its progression. Additionally, immune cell infiltration analysis and the TFs-DEG network revealed not only immune dysregulation but also transcriptional regulation in IPAH.

## Conclusion

IPAH is a disease entity with an unknown cause and is not a singular, homogenous disease in the traditional sense. This study identified ten hub genes, three TFs, and one biomarker of IPAH, confirmed in the PAH rat lung. The biological functions of DEGs and the TF-DEGs network contribute to a better understanding of the pathogenesis of IPAH. Meanwhile, we proposed that a dysregulated immune response was associated with chronic viral infection in IPAH lung tissue. Moreover, the immune infiltration profile may provide deep insights for IPAH immunotherapy. The relationship between MAPK6 and immune infiltration suggested a new immune treatment target for IPAH.

## Data Availability

The original contributions presented in the study are included in the article/Supplementary Material, further inquiries can be directed to the corresponding author.
